# Adiponectin Ameliorates Endotoxin-Induced Acute Cardiac Injury

**DOI:** 10.1155/2014/382035

**Published:** 2014-08-10

**Authors:** Yoshio Watanabe, Rei Shibata, Noriyuki Ouchi, Takahiro Kambara, Koji Ohashi, Li Jie, Yoko Inoue, Toyoaki Murohara, Kimihiro Komori

**Affiliations:** ^1^Department of Vascular Surgery, Cardiology, Nagoya University Graduate School of Medicine, Nagoya 466-8550, Japan; ^2^Department of Cardiology, Nagoya University Graduate School of Medicine, 65 Tsurumai, Showa, Nagoya 466-8550, Japan; ^3^Molecular Cardiovascular Medicine, Nagoya University Graduate School of Medicine, Nagoya 466-8550, Japan

## Abstract

*Background*. Obesity is a risk factor for cardiovascular disease. Increasing evidence suggests that reduced levels of the adipocyte-derived plasma protein adiponectin are associated with an increased cardiovascular risk. Here, we examined the effects of adiponectin on lipopolysaccharide- (LPS-) induced acute cardiac injury *in vivo*. *Methods and Results*. A single dose of LPS (10 mg/kg) was intraperitoneally injected into wild-type (WT) and adiponectin-knockout (APN-KO) mice. Following LPS administration, APN-KO mice had exacerbation of left ventricular (LV) systolic dysfunction compared with WT mice. Administration of LPS to WT and APN-KO mice led to an increased expression of inflammatory cytokines including TNF-*α* and IL-6 in the heart, but the magnitude of this induction was greater in APN-KO mice compared to WT mice. Systemic delivery of an adenoviral vector expressing adiponectin (Ad-APN) improved LPS-induced LV dysfunction in APN-KO mice, and this effect was accompanied by the reduced expression of TNF-*α* and IL-6 in the heart. Administration of etanercept, a soluble TNF receptor abolished the reduced LV contractile function in response to LPS in APN-KO mice. *Conclusion*. These results suggest that adiponectin protects against LPS-induced acute cardiac injury by suppressing cardiac inflammatory responses, and could represent a potential therapeutic target in sepsis-associated myocardial dysfunction.

## 1. Introduction

Septic shock is a serious complication and remains one of major causes of death in industrialized countries [1]. Cardiac contractile dysfunction is a common feature of endotoxemia in patients [2–4] and is also observed in experimental animal models of lipopolysaccharide- (LPS-) induced sepsis [5–7]. The presence of endotoxin in the blood stream is a primary contributor of septic shock, and several proinflammatory cytokines such as TNF-*α* and IL-6 can contribute to cardiac dysfunction during sepsis [5, 6].

Obesity affects the development and outcome of sepsis [8, 9]. Morbidly obese patients show prolonged mechanical ventilation time, longer weaning periods, and higher intensive care unit (ICU) mortality than leaner counterparts, and ICU complications, including sepsis, frequently occur in these patients [8, 9]. Furthermore, extremely obese patients undergoing emergent surgery develop sepsis and septic shock more often than normal weight patients [10], and it has been shown that sepsis-induced proinflammatory states correlate with the amount of total body fat [11–13].

Adipose tissue produces various secretory proteins, also known as adipocytokines [14, 15]. Obesity leads to the imbalance of adipocytokine production, resulting in the development of obesity-related metabolic and cardiovascular diseases [14, 15]. Adiponectin is an adipocytokine whose levels are decreased in association with cardiovascular risk factors such as type 2 diabetes, hypertension, dyslipidemia, and low-grade inflammation [16, 17]. Consistent with these clinical findings, experimental studies have shown that adiponectin deficiency contributes to diet-induced insulin resistance in connection with increased TNF-*α* levels, hypertension, and vascular dysfunction. Conversely, adiponectin enhances insulin sensitivity and reduces inflammatory reactions in vascular endothelial cells [16, 17]. Thus, adiponectin plays a protective role in obesity-inducible metabolic and vascular complications.

A number of animal studies showed that adiponectin has a protective effect against the development of various heart diseases. We have previously demonstrated that adiponectin-deficient (APN-KO) mice develop larger infarcts in the heart following ischemia-reperfusion injury [18], and that adiponectin administration leads to reduced myocardial injury and improved function following ischemia-reperfusion in mice and pigs [18, 19]. Ablation of adiponectin also causes concentric cardiac hypertrophy following pressure overload in mice [20]. Moreover, adiponectin has been shown to inhibit the development of doxorubicin-induced cardiomyopathy, which is the serious complication after the long-term use of this agent for cancer treatment [21]. However, little is known about the role of adiponectin in regulation of sepsis-associated cardiac dysfunction. In the present study, we evaluated the effect of adiponectin on LPS-induced cardiac inflammation and left ventricular (LV) dysfunction in wild-type and APN-KO mice.

## 2. Methods

### 2.1. Materials

LPS was purchased from Calbiochem (San Diego, CA, USA). Etanercept, a soluble TNF receptor, was purchased from Amgen (Thousand Oaks, CA, USA). Adenoviral vectors containing the gene for *β*-galactosidase (Ad-*β*gal) and full-length mouse adiponectin (Ad-APN) were prepared as described previously [20].

### 2.2. Animals and Experimental Model

Male wild-type (WT) (The Jackson Laboratory) and adiponectin-knockout (APN-KO) mice on a C57BL/6J background at 8 to 10 weeks of age were used in this study. Mice were intraperitoneally injected with a single dose of LPS (10 mg/kg) or PBS as described previously [6]. In some experiments, 2 × 10^8^ plaque-forming units (pfu) of Ad-APN or Ad-*β*gal were systemically injected into the tail vein of mice 3 days before LPS injection. Heart rate and blood pressure were determined using a tail-cuff pressure analysis system (Softron; Tokyo, Japan). In other experiments, etanercept, a soluble TNF receptor (8 mg/kg) or vehicle was given by intraperitoneal injection in mice 1 day before LPS treatment [22]. The study protocol was approved by the Institutional Animal Care and Use Committee of Nagoya University School of Medicine.

### 2.3. Echocardiographic Analysis

Surviving mice were subjected to transthoracic echocardiography to evaluate cardiac structure and function in the conscious state 6 hours following LPS injection. Echocardiogram analysis was performed to measure left ventricular (LV) systolic function and chamber dimensions, using an Acuson Sequoia C-256 machine with a 15 MHz probe. We quantified LV end systolic diameter (LVDs), LV end diastolic diameter (LVDd), and %LV fractional shortening (%FS) from M-mode images [21].

### 2.4. Measurement of mRNA

Total RNA from cultured cells was prepared using a RNA isolation kit (Qiagen; Valencia, CA, USA) according to manufacturer's protocols. Complementary DNA (cDNA) from 500 ng of total RNA was synthesized by reverse transcription using the SuperScript RT-PCR System (Invitrogen) according to manufacturer's instructions. Quantitative real-time RT-PCR (qRT-PCR) analysis was performed on a CFX-96 system using EvaGreen as a double-stranded DNA-specific dye according to the manufacture's instruction (Bio-Rad; Hercules CA, USA). Primers were designed as follows: 5′-ACCACCATCAAGGACTC-3′ and 5′-TGACCACTCTCCCTTTG-3′ for mouse TNF-*α*; 5′-TTCCAATGCTCTCCTAACAG-3′ and 5′-CTAGGTTTGCCGAGTAGATC-3′ for mouse IL-6; 5′-TCCTTCTTGGGTATGGAATC-3′; 5′-TAGAGGTCTTTACGGATGTC-3′ for *β*-actin. The expression levels of examined transcripts were compared to that of *β*-actin and normalized to the mean value of controls.

### 2.5. Statistical Analysis

All analyses were performed using PASW Statistics18 software (SPSS Inc., IL, USA). The student *t*-test was performed for comparison between two groups, and the two-way ANOVA test was used for comparison among multiple groups. All data are shown as mean ± SE, and significance was established at *P* < 0.05.

## 3. Results

### 3.1. APN-KO Mice Had Enhanced Cardiac Dysfunction following LPS Injection

**Figure 1:**
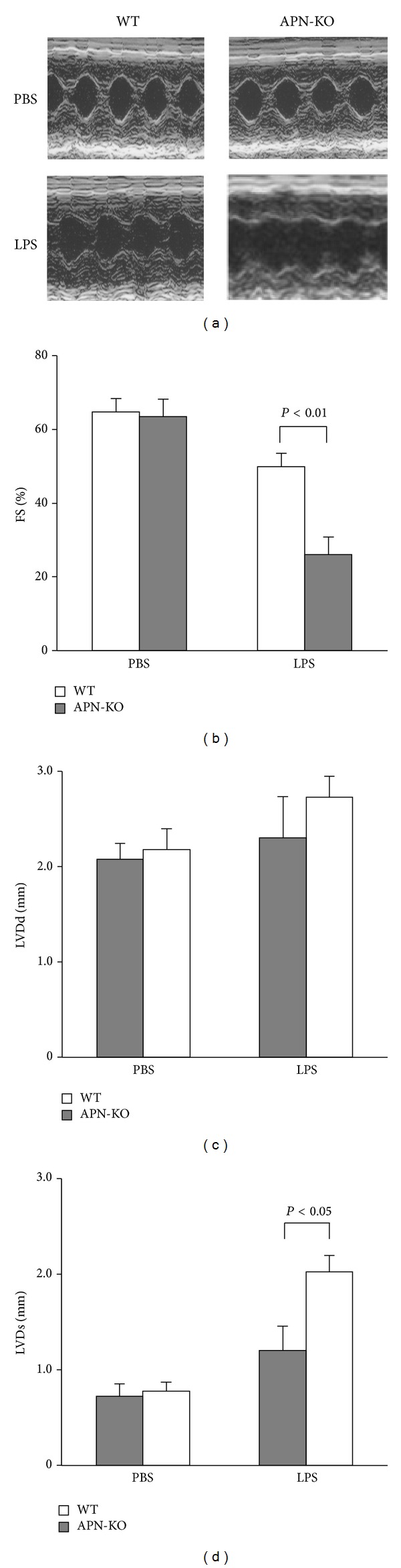
Loss of adiponectin results in exacerbated LPS-induced cardiac dysfunction. (a) Representative M-mode echocardiograms for WT and APN-KO mice 6 h after LPS or control vehicle injection. (b)–(d) Quantitative analysis of the fractional shortening (FS) (b), LV end diastolic dimension (LVDd) (c), and LV end systolic dimension (LVDs) (d), in WT and KO mice 6 h after LPS or vehicle injection (*n* = 5 in each group). Results are presented as mean ± SE.

To investigate the effect of adiponectin on sepsis-induced cardiac dysfunction, we intraperitoneally injected a single dose of LPS (10 mg/kg) or vehicle into APN-KO or WT mice.  Figure 1(a) shows representative M-mode echocardiograms for APN-KO and WT mice at 6 hours after LPS injection. Echocardiographic analysis showed that LPS injection led to an increase in LVDs and a decrease in % LV fractional shortening (%FS) in both APN-KO and WT mice without affecting LVDd. APN-KO mice showed increased LVDs and decreased %FS compared to WT mice following LPS injection (Figures 1(b)–1(d)). LVDd did not differ between APN-KO and WT mice following LPS injection, and there were no significant differences in LVDd, LVDs, or %FS between APN-KO and WT mice after injection of vehicle control (Figures 1(b)–1(d)).

### 3.2. Elevated Expression of Inflammatory Cytokines in APN-KO Mice following LPS Injection

**Figure 2:**
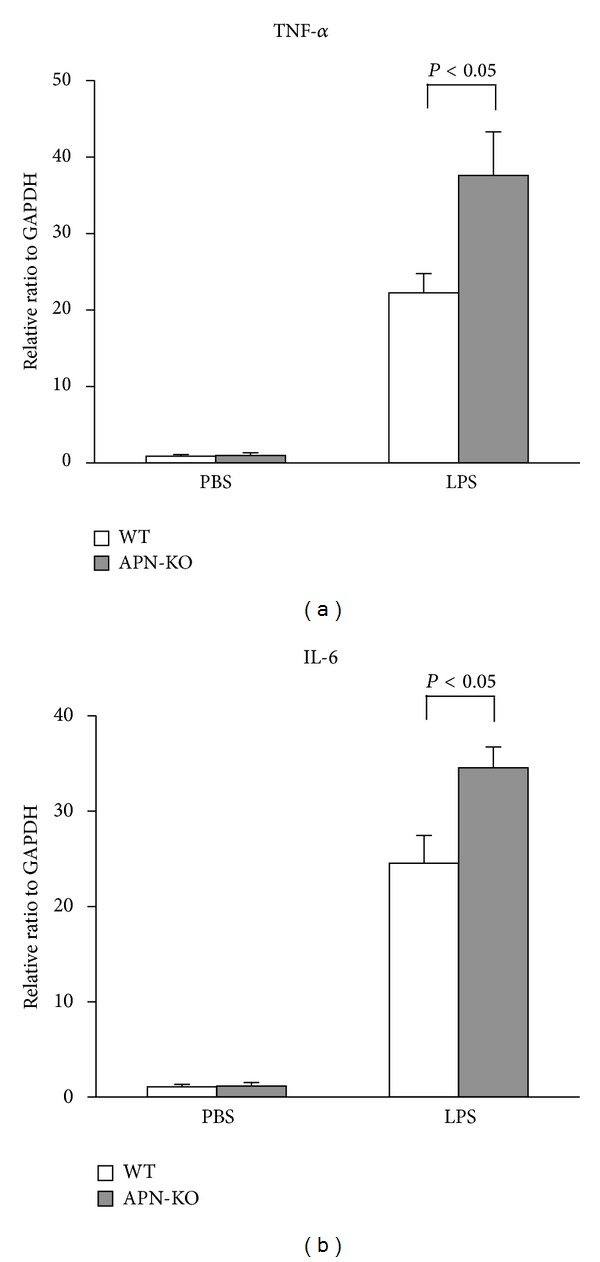
Increased cardiac inflammatory cytokines following LPS administration in APN-KO mice. (a) Myocardium TNF-*α* levels in WT (*n* = 5) and APN-KO (*n* = 5) mice 6 h after LPS or vehicle injection. (b) Myocardium IL-6 levels in WT (*n* = 5) and APN-KO (*n* = 5) mice 6 h after LPS or vehicle injection. Levels of mRNA in the myocardium of WT and APN-KO mice were quantified by real-time RT-PCR and expressed relative to GAPDH mRNA levels. Results are presented as mean ± SE.

Because inflammation contributes to cardiac dysfunction during sepsis, TNF-*α* and IL-6 mRNA levels in the myocardium were assessed by real-time PCR 6 hours following LPS administration in APN-KO and WT mice. Consistent with previous reports [6, 23], LPS led to increased myocardial TNF-*α* and IL-6 mRNA levels in WT mice (Figures 2(a) and 2(b)). Increased TNF-*α* and IL-6 mRNA levels were also observed in APN-KO mice, and the magnitudes of these increases were greater compared to WT (Figures 2(a) and 2(b)). There were no significant differences in cardiac TNF-*α* or IL-6 levels between APN-KO and WT mice following the injection of vehicle control (Figures 2(a) and 2(b)).

### 3.3. Adiponectin Supplementation Leads to Improvements in LPS-Induced Cardiac Dysfunction in WT and APN-KO Mice

**Figure 3:**
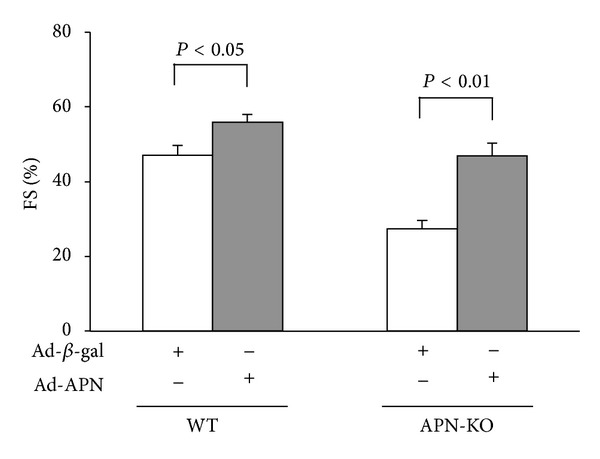
Adenoviral expression of adiponectin improves LPS-induced cardiac dysfunction. Quantitative analysis of %FS 6 h following LPS injection in WT and APN-KO mice pretreated with Ad-APN or Ad-*β*gal (control). Ad-APN or Ad-*β*gal (2 × 10^8^ pfu total) was delivered intravenously via the tail vein 5 d before LPS injection (*n* = 5 in each group). Results are presented as mean ± SE.

To assess whether adiponectin modulates LPS-mediated LV contractile dysfunction, we systemically treated APN-KO and WT mice with adenoviral vectors expressing either adiponectin (Ad-APN) or *β*gal (Ad-*β*gal) via tail vein injection 5 days prior to LPS induction. At the time of LPS administration, adiponectin levels were 22.1 ± 4.3 *μ*g/mL in WT treated with Ad-APN, 11.8 ± 2.0 *μ*g/mL in WT treated with control, 12.4 ± 2.5 *μ*g/mL in APN-KO treated with Ad-APN, and less than 0.05 *μ*g/mL in APN-KO treated with control. Both WT and APN-KO mice receiving Ad-APN showed significantly increased %FS following LPS injection, as compared to WT or APN-KO mice treated with the Ad-*β*gal control vector (Figure 3).

### 3.4. TNF-*α* is Involved in the Protective Effect of Adiponectin on LPS-Induced Cardiac Dysfunction

**Figure 4:**
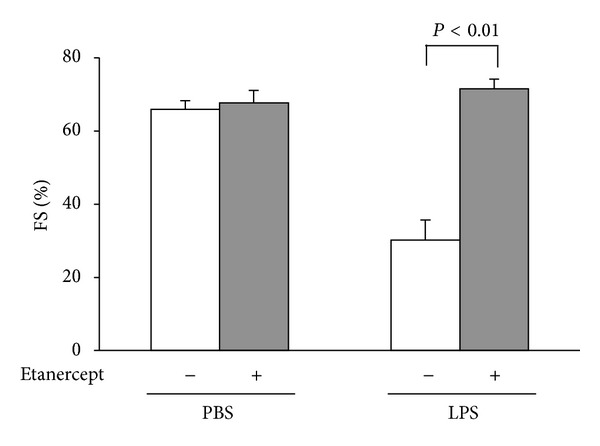
Neutralization of TNF-*α* ameliorates LPS-induced cardiac damage in APN-KO mice.Quantitative analysis of %FS following treatment with etanercept, a soluble TNF receptor or vehicle, in APN-KO mice 6 h after LPS injection (*n* = 5). Etanercept (8 mg/kg) or vehicle was given by intraperitoneal injection in APN-KO mice 1 d before LPS treatment. Results are presented as mean ± SE.

To analyze the involvement of TNF-*α* in the cardio-protective effect of adiponectin* in vivo*, APN-KO mice were treated with either etanercept or vehicle by intraperitoneal injection followed by stimulation with LPS. Treatment with etanercept attenuated the LPS-induced %FS reduction as compared to vehicle in APN-KO mice (Figure 4). These data suggest that the elevated cardiac TNF-*α* production following LPS treatment contributes to cardiac dysfunction observed in APN-KO mice.

## 4. Discussion

Results from this investigation suggest that adiponectin confers resistance to myocardial damage in an animal model of LPS-induced sepsis by suppressing cardiac inflammation. APN-KO mice showed greater LV contractile dysfunction following LPS administration compared to WT mice, and adenoviral delivery of adiponectin improved LPS-induced LV dysfunction in both APN-KO and WT.

The release of LPS, the major outer membrane component of Gram-negative bacteria, induces a dysregulated immune response characterized by the overproduction of TNF-*α* and IL-6 [24]. Studies have shown that mice deficient in TNF-*α* exhibit less cardiac damage [25], and treatment with a soluble TNF receptor or anti-TNF-*α* antibody limits the damage caused by acute myocardial injury such as LPS administration [26–28]. Our group previously reported that adiponectin inhibits LPS-induced TNF-*α* production in cardiac myocytes and fibroblasts [18]. Ischemia-reperfusion in APN-KO mice also result in increased myocardial TNF-*α* expression [18]. Supplementation of adiponectin diminishes infarct size with associated reductions in myocardial TNF-*α* production in APN-KO and WT mice [18]. Adiponectin was also shown to reduce LPS-stimulated TNF-*α* production and increase anti-inflammatory cytokine IL-10 levels in human macrophages [29, 30]. Thus, adiponectin exerts anti-inflammatory actions in various types of cells, leading to protection against the progression of inflammatory diseases.

In this study, APN-KO mice showed markedly higher TNF-*α* levels in heart tissue following LPS injection compared to WT. Furthermore, treatment with etanercept, a soluble TNF receptor reduced LPS-induced cardiac damage in APN-KO mice. These data indicate that the protective action of adiponectin against LPS-induced myocardial damage is mediated, at least in part, by its ability to suppress upregulation of TNF-*α* in the heart. Similarly, it has been shown that the ability of adiponectin to attenuate retinal vessel injury during hypoxia is largely dependent on its ability to suppress TNF inflammatory response [31].

Numerous clinical studies have reported a positive correlation between mortality and obesity in the medical ICU. Experimental studies showed that cerebral venules of mice after perforation assumed a proinflammatory and prothrombogenic phenotype, with greatly exaggerated responses in obese (ob/ob) mice [32]. A more prominent inflammatory response to cecal ligation and puncture- (CLP-) induced sepsis has also been observed in the intestinal microcirculation of obese mice (ob/ob and db/db) [33]. This obesity related to exaggerated sepsis-induced tissue injury response is linked to inflammation, and obesity-related disorders are well known to be associated with low levels of adiponectin. Data presented here show that adiponectin protects against LPS-induced acute cardiac injury by suppressing cardiac inflammation. Our results also suggest that adiponectin functions are a crucial adipocytokine that affects cardiac function and could be associated with the prognosis in patients with sepsis. Adiponectin exhibits anti-inflammatory properties, and adiponectin supplementation could therefore be beneficial for the treatment or prevention of inflammatory diseases.

## Abbreviations

APN: Adiponectin
LPS: Lipopolysaccharide
LV: Left ventricular
KO: Knockout.
